# A comparative study of functioning and non-functioning pituitary adenomas

**DOI:** 10.1097/MD.0000000000025306

**Published:** 2021-04-09

**Authors:** Jiayin Qin, Kai Li, Xijuan Wang, Yongzhen Bao

**Affiliations:** a Department of Ophthalmology, Peking University People's Hospital, Eye Diseases and Optometry Institute, Beijing Key Laboratory of Diagnosis and Therapy of Retinal and Choroid Diseases, College of Optometry, Peking University Health Science Center, Xi Cheng District; b Department of Ophthalmology; c Department of Neurosurgery, Peking University International Hospital, Life Science Park of Zhong Guancun, Chang Ping District, Beijing, China.

**Keywords:** clinical characteristics, functioning pituitary adenoma, non-functioning pituitary adenoma, visual function impairment

## Abstract

Pituitary tumors commonly cause visual impairment and the degree of impairment can depend on the size, location, and type of the tumor. However, no studies have been made regarding the differences caused by functioning pituitary adenoma (FPA) and non-functioning pituitary adenoma (NFPA). We aimed to investigate the relationship between clinical characteristics and visual impairment in patients with FPA and NFPA.

This case series study included 73 pituitary adenoma patients. All patients underwent ophthalmic evaluations, and we retrospectively reviewed their medical records. Tumor types were confirmed by histological analysis, and the tumor volume was calculated. Magnetic resonance imaging was used to determine the tumor diameter. The observation indices of the two groups were compared. The correlation between the visual field and tumor volume was analysed using scatter plots.

We enrolled 30 patients in the FPA group and 43 in the NFPA group. The first symptoms presented in the eyes in 23% of FPA patients and 41.9% of NFPA patients. The best-corrected visual acuity of the FPA group was better than that of the NFPA group, and 34 (56.7%) and 73 (84.9%) eyes in these groups had visual field defects, respectively. The visual field defects of the FPA patients were lighter than those of the NFPA patients. Except for the anteroposterior diameter, there were no differences in the other parameters of tumor diameter between the groups. The tumor volume of the FPA group was smaller than that of the NFPA group. The tumor size was positively correlated with the mean deviation and negatively correlated with the mean sensitivity in both groups.

There was a longer delay between the onset of signs and symptoms and treatment in the FPA group than in the NFPA group. Future studies should focus on visual field defects caused by FPA and NFPA.

## 1 Introduction

The pituitary gland is formed from the adenohypophysis (anterior pituitary) and the neurohypophysis (posterior pituitary) and lies in the sella turcica, with the cavernous sinus on both sides and the optic chiasmata above.^[1]^ The adenohypophysis wraps around the front and both sides of the neurohypophysis in the shape of a “V” and comprises ∼80% of the volume of the pituitary gland. It secretes thyrotropin, adrenocorticotropic hormone, gonadotropin, and growth hormone. In contrast, the neurohypophysis accounts for the remaining 20% of the pituitary gland volume; it is responsible for storing and releasing vasopressin and oxytocin, which are hormones secreted by the hypothalamus.

Pituitary adenomas are a common tumor type which develop in the brain. According to a recent study, 15.5% of central nervous system tumors are pituitary adenomas. Gliomas comprise 15.6% and meningiomas comprise 37.1% of central nervous system tumors.^[2]^ Pituitary adenomas are classified based on their functions, which include the synthesis and secretion of bioactive hormones. Non-functioning pituitary adenoma (NFPA) and functioning pituitary adenoma (FPA) are the two main types of pituitary adenomas; furthermore, FPAs are divided into prolactinomas, growth hormone tumors, adrenocorticotrophic hormone tumors, thyroid hormone tumors, gonadotropic hormone tumors, and multi-hormone adenomas. FPAs are usually treated for systemic symptoms, which develop due to the excessive secretion of hormones. In contrast, NFPAs are adenomas with no clinical evidence of hormone hypersecretion. These tumors usually present with the effects of local pressure, such as visual disturbances, headaches, and decreases in pituitary hormones.^[3]^ Many studies have confirmed that symptoms of visual function impairment caused by pituitary tumors may vary depending on the type, size, location, and rate of growth of the tumors.^[4]^ However, there are no known studies reporting the differences in visual function impairment caused by FPAs and NFPAs.

In this study, we assessed the medical records of patients who underwent surgery for pituitary adenomas. We identified the clinical features of patients with NFPA or FPA and analyzed differences in visual function impairment between the two types.

## 2 Materials and methods

### 2.1 Patients and ethical considerations

We retrospectively reviewed the medical records of patients with pituitary adenoma who underwent endoscopic endonasal or transcranial approach surgery in our hospital between September 2016 and December 2019 and requested ophthalmic consultations. Tumor types were confirmed using histological analysis in all patients.

We included patients who

1. had primary pituitary adenomas that were confirmed using magnetic resonance imaging (MRI),
2. had available preoperative data records of pituitary adenomas (three plane sizes), and
3. underwent ophthalmic examinations, which included vision, intraocular pressure, funduscopic examination, and Octopus visual field examinations.

In contrast, we excluded patients who:

1. had any other ophthalmic or systemic comorbidities that could affect visual function, such as cataracts, glaucoma, retinopathy, or acute cerebral infarction, or
2. had nervous system symptoms caused by tumors that made them unable to comply during ophthalmic examinations.

The study protocol was reviewed and approved by the Ethical Committee of the Peking University International Hospital. This study was performed in accordance with the Declaration of Helsinki for biomedical research involving human patients. Written informed consent was obtained from each patient after an explanation of the risks, benefits, and alternatives of the study.

### 2.2 Ophthalmological and neurological evaluations

Basic information, including the name, sex, age, first symptoms, and duration of symptoms were recorded for all patients. Furthermore, all patients were required to undergo a preoperative assessment, which evaluated the best-corrected visual acuity (BCVA), intraocular pressure, fundus, and the visual field (using visual standard automated perimetry [HAAG-STREIT, Octopus900]). We classified patients based on the characteristics of visual field deficiency that were observed. The tumor diameter was measured and recorded for three dimensions, while the tumor volume (Cavalieri's principle) was calculated using the following formula:


$$\text{tumour}\;\text{volume}=\frac{4}{3}\pi *\frac{a}{2}*\frac{b}{2}*\frac{c}{2}\text{c}{\text{m}}^{3}$$

*a*, *b*, and *c* represent the diameters measured in the three dimensions of the tumor, respectively.^[5]^

According to the results of the postoperative pathological and immunohistochemical examinations, patients were divided into two groups:

1. the FPA group and
2. the NFPA group.

The sex ratio, age, duration of symptoms, BCVA, tumor size (the diameter of three dimensions and the volume of the tumor), visual field parameters (including mean deviation [MD] and mean sensitivity [MS]), and visual field deficiency of the two groups were compared. The correlations between the visual field parameters and tumor volume of the two groups were determined, and a scatter plot was drawn.

### 2.3 Statistical analyses

The demographic and clinical data were summarized using standard descriptive statistics (means ± standard deviation [SD]) and frequency tabulation (%). The chi-squared test was conducted to compare nonparametric values. For other indices, the independent two-sample *t* test or Mann–Whitney *U* test is used. *P* < .05 was considered statistically significant, and all *P*-values were two-tailed (SPSS Statistics version 24, IBM).

## 3 Results

### 3.1 Patient characteristics

Table 1 displays the characteristics for all patients included in this study. In total, 146 eyes of 73 patients were included in the analysis. Among them, 35 (47.9%) were male, 38 (52.1%) were female, and the age ranged between 15 and 74 years (median value: 50; mean ± SD: 48.41 ± 13.36). Furthermore, the shortest time between onset of signs and symptoms of FPA/NFPA and diagnosis was 7 days, while the longest time was 2160 days; the median value of onset was 180 days, and the average time of onset was 374.61 days. The first symptoms included visual acuity decline, visual field defects, and other ocular manifestations in 32.9% of patients (48 eyes of 24 patients); headache, dizziness, and other nervous system manifestations in 30.1% of patients (44 eyes of 22 patients); and acromegaly, amenorrhea, lactation, and other endocrine system manifestations in 20.5% of patients (30 eyes of 15 patients). A total of 16.4% of patients (24 eyes of 12 patients) reported no signs or symptoms consistent with a pituitary adenoma. Signs and symptoms of a pituitary adenoma were identified incidentally during routine examinations.

**Table 1:** Patient characteristics and clinical findings.

Variable	Value
Number of patients	73
Male:Female	35 (47.9%):38 (52.1%)
Age at diagnosis (years, median value)	15–75 (50)
Initial symptom (cases)	
Ophthalmological	25 (34.2%)
Neurological	21 (28.8%)
Endocrine	16 (21.9%)
Physical examination	11 (15.1%)
BCVA	0.70 ± 0.37
Visual field parameter (dB)	
MD	11.57 ± 8.39
MS	16.49 ± 8.48
Tumor size	
Transverse diameter (cm)	2.40 ± 0.99
Anteroposterior diameter (cm)	2.05 ± 0.96
Craniocaudal diameter (cm)	2.22 ± 1.02
Volume (cm^3^)	8.17 ± 9.37

BCVA = best correct visual acuity, MD = mean deviation, MS = mean sensitivity.

### 3.2 Comparison of characteristics and clinical findings between the FPA and NFPA groups

Table 2 presents the clinical findings and comparisons between the groups. There were 11 male and 19 female patients in the FPA group, and 23 male and 20 female patients in the NFPA group. Among the FPA group, there were 10 cases of Somatotroph adenoma (33.33%), 7 cases of corticotroph adenoma (23.33%), 7 cases of lactotroph adenoma (23.33%), 4 cases of plurihormonal adenoma (13.33%), and 2 cases of gonadotroph adenoma (6.67%). The median age of the FPA group (44 years) was significantly lower than that of the NFPA group (53 years) (*P* < .001). In 23% of the FPA group and 41.9% of the NFPA group, the first symptoms occurred in the eyes; the difference between the groups was statistically significant (χ^2^ = 10.671, *P* = .014). The anteroposterior diameter of the tumor was significantly smaller in the FPA group (*P* = .013). There was no significant difference in the transverse or craniocaudal tumor diameter between the two groups. Furthermore, the mean tumor volume of the FPA group (6.16 ± 6.52 cm^3^) was significantly smaller than that of the NFPA group (9.62 ± 10.64 cm^3^) (*t* = −2.088, *P* = .039).

**Table 2:** Comparison of characteristics and clinical findings between patients with functioning and non-functioning pituitary adenomas.

Variable	FPA (n=30)	NFPA (n = 43)	*P*
Sex			
Male	12 (16.4%)	23 (31.5%)	.315
Female	18 (24.7%)	20 (27.4%)	–
Age (years, median value)	44	53	0.000^∗∗^
Duration of symptoms (days, median value)	360	120	.005^∗^
Initial symptom			
Ophthalmological	7 (23.3%)	18 (41.9%)	.014^∗^
Neurological	3 (10%)	18 (41.9%)	–
Endocrine	16 (53.3%)	0	–
Health examination	4 (13.3%)	7 (16.2%)	–
Tumor size			
Transverse diameter (cm)	2.27 ± 0.92	2.52 ± 1.04	.155
Anteroposterior diameter (cm)	1.80 ± 0.73	2.22 ± 1.05	.013^∗^
Craniocaudal diameter (cm)	2.05 ± 0.97	2.35 ± 1.04	.100
Volume (cm^3^)	6.16 ± 6.52	9.62 ± 10.64	.039^∗^

FPA = functioning pituitary adenoma, NFPA = non-functioning pituitary adenoma. ^∗^*P* < .05; ^∗∗^*P* < .001.

### 3.3 Comparison of visual function impairment between the FPA and NFPA groups

**Figure 1:**
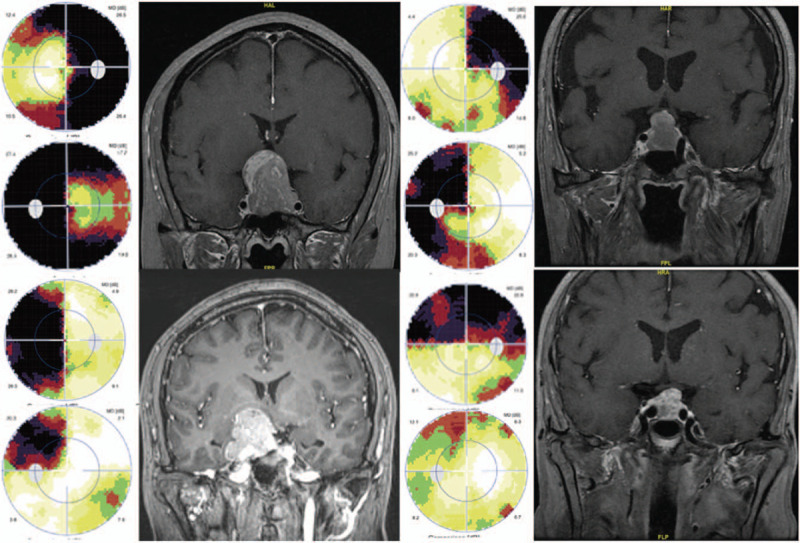
Visual field and MRI of four patients with pituitary adenoma. Top-left: Case 1. A 53-year-old woman with NFPA at diagnosis. Visual field: bilateral temporal hemianopsia breaking through the vertical midline. Contrast-enhanced MRI shows that the pituitary dumbbell-shaped mass with a clear boundary is significantly enhanced, with a size of 2.7 × 2.6 × 4.0 cm. The mass protrudes to the sella and compresses the optic chiasma. Top-right: Case 2. A 70-year-old man with NFPA at diagnosis. Visual field: bilateral superior temporal quadrant visual field defect and breakthrough to the inferior temporal region. Contrast-enhanced MRI shows that the tumor is significantly enhanced, with a size of 1.9 × 1.8 × 2.5 cm and a clear boundary. The pituitary stalk is shifted to the left, and the optic chiasma is displaced upwards. Bottom-left: Case 3. A 47-year-old man with FPA (parasellar prolactinoma) at diagnosis. Visual field: nasal hemianopsia in the right eye and a quadrant visual field defect in the left eye. Contrast-enhanced MRI shows that the tumor is significantly enhanced, with a size of 2.0 × 2.7 × 3.0 cm and an irregular boundary. The pituitary stalk is shifted to the left, and the anterior part of the optic chiasma is obviously elevated on the right side. Bottom-right: Case 4. A 67-year-old man with NFPA at diagnosis. Visual field: superior hemianopsia in the right eye and a superior local defect in the left eye. Contrast-enhanced MRI shows that the tumor is 1.8 × 1.7 × 1.9 cm, and the left optic chiasma is compressed and displaced upwards. MRI = magnetic resonance imaging, FPA = functioning pituitary adenoma, NFPA = non-functioning pituitary adenoma.

The BCVA of the FPA group (0.81 ± 0.30) was significantly higher than that of the NFPA group (0.61 ± 0.39) (*t* = 2.930, *P* = .004). When comparing the MD and MS, the degree of visual field defects in the FPA group was lighter than that of the NFPA group, and both parameters were significantly different between the groups (MD: *t* = −3.082, *P* = .002; MS: *t* = 3.448, *P* = .001). Thirty-four eyes (56.7%) of 19 patients and 73 eyes (84.9%) of 40 patients in the FPA and NFPA groups, respectively, had visual field defects, and the difference between the groups was statistically significant (χ^2^ = 54.822, *P* < .001). The types of visual field defects in the patients are summarized in Table 3. Among the abnormal defects, most visual field defects were temporal hemianopsia. Among the patients with these defects, 79.17% in the FPA group and 94.74% in the NFPA group did not break through the vertical midline. Temporal hemianopsia was followed by hemianopia with the horizontal midline as a boundary. Interestingly, there were 2 patients in the FPA group and 3 in the NFPA group with nasal defects. Several examples of typical and atypical visual field defects are shown in Figure 1.

**Table 3:** Comparison of visual function impairment between patients with functioning and non-functioning pituitary adenomas.

Variable	FPA (n = 30)	NFPA (n = 43)	*P*
BCVA	0.81 ± 0.30	0.61 ± 0.39	.004^∗^
VF parameter (dB)			
MD (dB)	8.87 ± 6.98	13.33 ± 8.82	.002^∗^
MS (dB)	19.55 ± 7.06	14.54 ± 8.83	.001^∗∗^
Types of VF manifestations			
Normal	26 (43.3%)	17 (19.8%)	0.000^∗∗^
Temporal hemianopsia not breaking through the vertical midline	19 (31.7%)	35 (40.7%)	–
Temporal hemianopsia breaking through the vertical midline	5 (8.3%)	3 (3.5%)	–
Hemianopia with the horizontal midline as a boundary	3 (5.0%)	7 (8.1%)	–
Nasal defect	2 (3.3%)	5 (5.8%)	–
Others	5 (8.3%)	19 (22.1%)	–

BCVA = best-corrected visual acuity, FPA = functioning pituitary adenoma, MD = mean deviation, MS = mean sensitivity, NFPA = non-functioning pituitary adenoma, VF = visual field. ^∗^*P* < .05 and ^∗∗^*P* < .001.

### 3.4 Correlation between the tumor size and visual field parameters

**Figure 2:**
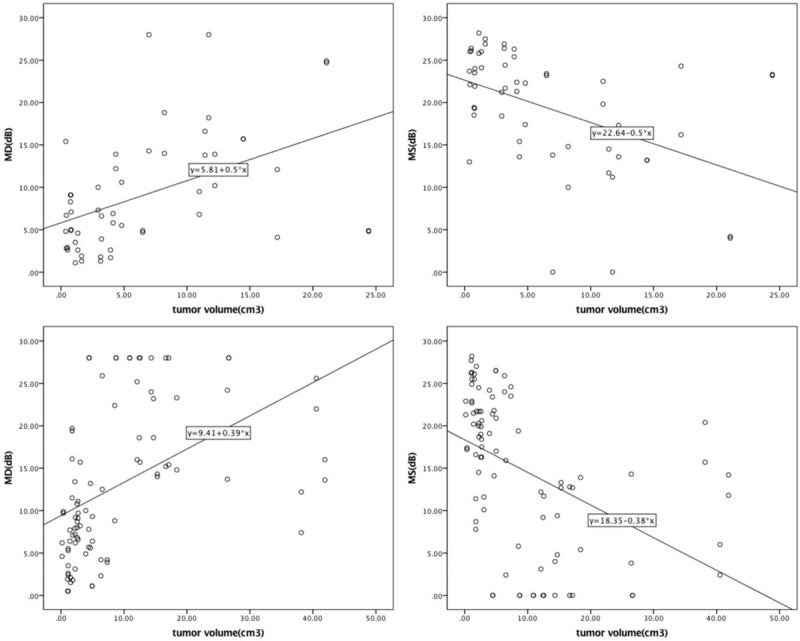
Correlation between the tumor size and visual field parameters. Top-left: Correlation between the mean deviation (MD) and tumor volume in patients in the FPA group. Pearson correlation coefficient = 0.515, *P* < .001. Top-right: Correlation between the mean sensitivity (MS) and tumor volume in patients of the FPA group. Pearson correlation coefficient = −0.512, *P* < .001. Bottom-left: Correlation between the MD and tumor volume in patients of the NFPA group. Pearson correlation coefficient = 0.446, *P* < .001. Bottom-right: Correlation between the MS and tumor volume in patients of the NFPA group. Pearson correlation coefficient = −0.437, *P* < .001. FPA = functioning pituitary adenoma, NFPA = non-functioning pituitary adenoma.

We analyzed the correlation between the tumor size and the visual field parameters and compared these values between the two groups. It was found that the tumor size was significantly and positively correlated with the MD (*P* < .001) and negatively correlated with the MS (*P* < .001) in both groups. The correlations between the visual field parameters (MD and MS) and tumor volume are shown in Figure 2.

## 4 Discussion

In this study, we analyzed the clinical data, including age, sex ratio, clinical manifestations, and tumor size, in patients with FPAs and NFPAs. The parameters such as age, sex ratio, and tumor size of the NFPA group were similar to those reported in a previous study.^[6]^ However, no known previous studies have compared the clinical characteristics of FPA and NFPA patients. Our study found that there was no significant difference in the sex distribution, and in the transverse and craniocaudal tumor diameter between the two groups. However, we found that mean age was significantly lower in the FPA group. Additionally, anteroposterior diameter and tumor volume were lower in the FPA group. These findings are consistent with a previous study that reported that NFPAs are more frequent among all giant pituitary adenomas.^[7]^

In 1978, a study reported that the main complaint of patients with pituitary adenoma was a decrease in visual function.^[8]^ Nowadays, with advancements allowing accurate testing for hormone detection and neuroimaging, the diagnosis of pituitary adenomas is made earlier. It has been previously reported that only 30% of patients presenting with pituitary tumors complain of visual problems.^[9]^ This rate is similar to that presented in our study. Meanwhile, the researchers have suggested that neurological manifestations are the initial symptoms of pituitary adenoma in less than 10% of cases.^[9]^ In our study, the FPA group demonstrated a similar rate of neurological manifestations, while the NFPA group had a higher proportion of these symptoms.

In terms of neuro-ophthalmological manifestations caused by a pituitary tumor, it is important to consider the anatomical structure of the pituitary gland. The pituitary gland is located in the dural sac of the sellar region, with cavernous sinuses on both sides. The optic chiasma is located above the pituitary gland. When the tumor grows upward, and its volume exceeds that of the seller space, the optic chiasma centre will be compressed; chronic compression of the optic chiasma by the tumor may result in axoplasmic stasis and hampered chiasmal blood supply, which may cause bilateral temporal hemianopsia and decreased vision. Furthermore, various patterns of visual field deficiency have been described in patients with pituitary adenomas, with the precise type of defect depending on the anatomy of the optic chiasma and its relationship to the tumor. The compression in the anterior angle of the optic chiasma produces temporal and superior visual field defects. Non-central tumors present as a combination of severe central visual loss in one eye and subtle defects in the superior temporal visual field, with respect to the vertical midline in the contralateral eye. Posterior lesions may involve the optic tracts, which leads to homonymous hemianopsia.^[10,11]^ In our study, the diversity of visual field deficiency was also present. The proportion of patients with normal visual fields in the FPA group was higher than that in the NFPA group. In the NFPA group, hemianopsia in the vertical middle line of the temporal side was the most common, while the quadrant defect in the upper or lower temporal side was the most common in the FPA group. However, atypical visual field defects were seen in both groups, including the upper or lower visual field defects bounded by the horizontal middle line and nasal quadrant defects.

Regardless of the presence of FPA or NFPA, when growth of the tumor increases to a certain extent, it will cause damage to visual functions.^[12,13]^ Before tumor growth, however, FPA may present with systemic symptoms related to excessive hormone secretion.^[9,14]^ It has also been reported that the diagnosis of NFPA is more delayed than that of FPA^[15,16]^; however, that report does not align with the findings of our present study. We found that there was a longer delay between the onset of signs and symptoms and treatment in the FPA group than in the NFPA group, which suggests that public awareness of the systemic symptoms caused by excessive secretion of such hormones is limited. Most of our patients did not pay attention to these signs and symptoms. Ignoring such signs and symptoms could lead to lifelong challenges such as compromised vision and headaches that could affect the patient's daily function and quality of life.

According to our results, the effect of FPA on visual function is relatively smaller than that of NFPA. Other scholars have studied the effect of pituitary tumor size on visual field parameters. Rivoal et al confirmed that pituitary tumor patients with visual field defects had larger tumor volumes; in that study, the only significant correlation was between the tumor size and the degree of visual field defects.^[17]^ Thomas et al also confirmed that the decrease in visual acuity and the degree of visual field defects were significantly related to tumor volume.^[18]^ Beltrame et al reported that the thickness of the retinal nerve fiber layer could be clearly detected by optical coherence tomography in patients with a large tumor volume; this is a critical finding because damage to this fiber layer can cause more serious damage to vision and the visual field.^[19]^ However, there are no known studies that have compared the effects of tumor volume on the visual field between FPA and NFPA patients. According to the relevant equation and scatter diagram, it can be found that the same volume of FPA causes less damage to the visual field than that of NFPA. This may be related to the growth pattern of NFPA because some invasive pituitary tumors were also included in the NFPA group.^[20]^ In addition, there is a mechanism of vascular dysfunction underlying visual function damage caused by pituitary tumors, which is more likely to occur in patients with NFPAs.^[21,22]^

This study has some limitations that should be addressed. First, because it is a retrospective study, rather than consecutive cases, only patients with pituitary adenomas who can complete ophthalmic examination and have complete data can be included in the study; therefore, there is an inherent selection bias that could not be avoided. Second, because many patients are transferred to our hospital from other places, the extent of disease is likely to be more serious and complex. Therefore, the long-term treatment effect should be observed and followed-up.

In conclusion, there was a longer delay between the onset of signs and symptoms and treatment in the FPA group than in the NFPA group. Furthermore, the tumor size was positively correlated with MD and negatively correlated with MS in both groups. Future studies should focus on multiple types of visual field defects caused by FPA and NFPA.

## Acknowledgments

We thank all the patients who participated in this study and for their willingness to contribute valuable data for this manuscript. We would like to thank Editage (www.editage.com) for English language editing.

## Author contributions

**Conceptualization:** Jiayin Qin, Yongzhen Bao.

**Data curation:** Jiayin Qin, Xijuan Wang, Yongzhen Bao.

**Formal analysis:** Jiayin Qin.

**Investigation:** Jiayin Qin, Xijuan Wang.

**Methodology:** Jiayin Qin, Kai Li, Yongzhen Bao.

**Supervision:** Kai Li, Yongzhen Bao.

**Validation:** Yongzhen Bao.

**Writing – original draft:** Jiayin Qin, Xijuan Wang.

**Writing – review & editing:** Kai Li, Yongzhen Bao.
